# Total and Differential Somatic Cell Count in Italian Local Cattle Breeds: Phenotypic Variability and Effect on Milk Yield and Composition

**DOI:** 10.3390/ani13071249

**Published:** 2023-04-04

**Authors:** Silvia Magro, Angela Costa, Massimo De Marchi

**Affiliations:** 1 Department of Agronomy, Food, Natural Resources, Animals and Environment, University of Padova, 35020 Padova, Italy; massimo.demarchi@unipd.it; 2 Department of Veterinary Medical Sciences, Alma Mater Studiorum University of Bologna, 40064 Bologna, Italy; angela.costa2@unibo.it

**Keywords:** Alpine Grey, Burlina, milk biomarker, neutrophil, intramammary inflammation

## Abstract

**Simple Summary:**

In addition to the historical indicator of mammary gland health, milk somatic cell count (SCC), the differential SCC (DSCC) has been introduced to improve the accuracy of mastitis detection. No studies have yet explored DSCC variability in local breeds used for milk production such as Burlina and Alpine Grey. Although local cattle breeds show greater rusticity and resistance to disease compared to cosmopolitan specialized dairy breeds, udder health status needs to be monitored for reasons related to profitability, management improvement, and animal welfare. In the present study, we aimed at investigating the factors affecting SCC and DSCC in Italian local breeds. Finally, by combining both SCC and DSCC, we attempted to estimate the effect of the udder health status on milk yield and composition traits.

**Abstract:**

Milk differential somatic cell count (DSCC) represents the percentage of polymorphonuclear neutrophils and lymphocytes out of the total somatic cell count (SCC) and has been proposed in recent years as a proxy for udder health in dairy cows. We investigated phenotypic factors affecting SCC and DSCC using 3978 records of 212 Alpine Grey and 426 Burlina cows farmed in Northern Italy. The linear mixed model accounted for the fixed effects of breed, parity, lactation stage, sampling season, and first-order interactions of breed with the other effects. Cow, herd-test-date nested within breed were random. Subsequently, four udder health status groups (UHS) were created by combining SCC and DSCC to assess the UHS impact on milk yield and quality. DSCC was greater in Alpine Grey (66.2 ± 0.8%) than Burlina cows (63.2 ± 0.6%) and, similarly to SCC, it increased with days in milk and parity regardless of breed. Milk yield and composition were affected by UHS in both breeds. These results suggest that also udder health of local breeds can be monitored on a large scale through SCC and DSCC for reduction in biodiversity loss and increased farm profitability. However, in addition to milk data, the introduction of mastitis recording and monitoring plans is advisable.

## 1. Introduction

In Italy, local cattle breeds are farmed for their contribution to a multifunctional and sustainable development and maintenance of confined areas. Representative examples of local breeds are the Burlina and the Alpine Grey, which are both well adapted to marginal environments where farming conditions can often be challenging [1,2]. Thanks to their good grazing aptitude, these dual-purpose breeds have been reared for both meat and milk production in extensive and semi-extensive systems. In this regard, the adaptability and the ability to transform poor pastures into valorized animal products make Burlina and Alpine Grey important genetic resources to preserve [1,2,3].

Generally, local cattle breeds show greater rusticity and resistance to disease compared to cosmopolitan genotypes specialized for dairy like Holstein [4]. Nevertheless, milk quality and udder health status need to be monitored in local as in cosmopolitan breeds for reasons related to profitability, management improvement, and animal welfare. Although enormous efforts have been put at different levels to improve udder health, mastitis is still the most impacting disease in cows [5]. In addition to the milk somatic cell count (SCC, cells/mL), historical indicator of mammary gland health, the differential somatic cell count (DSCC, %) has been recently introduced. The DSCC has been proposed as a promising novel trait to improve mastitis detection accuracy to be used as a proxy for management purpose (e.g., selective dry-cow therapy) [6,7,8]. In bovine milk the epithelial exfoliated cells account for approximately 10% of total SCC, with polymorphonuclear neutrophils, macrophages, and lymphocytes being the main components of SCC [9]. The DSCC is the fraction of SCC that includes polymorphonuclear neutrophils and lymphocytes [6,7,8]. Considering that the composition of SCC changes in the presence of inflammation, monitoring traits like DSCC in parallel with SCC sounds meaningful. In fact, SCC and DSCC observed during inflammation may differ from those observed after the onset of inflammation. For example, polymorphonuclear neutrophils are the predominant cell type in the early stages of inflammation and they decrease in chronically affected quarters [10,11]. Cows with one or more quarters suffering from a chronic mastitis are generally defined as ‘chronic’: these are expected to have a dampened immune response, particularly in presence of certain pathogens [12]. In fact, while the immune response activation occurs normally in healthy animals, a tolerance status can be observed in presence of chronic infections, i.e., attenuation of the inflammatory response, reduction in the concentration of inflammation biomarkers in both plasma and milk, and less evident or non-evident signs [12].

Few milk laboratories carrying out official milk analyses are equipped with devices able to record DSCC in Italy. Data are thereby routinely registered only in certain Italian regions. This explains why, to date, milk DSCC of Burlina and Alpine Grey has never been explored.

In the era of precision livestock farming, however, farmers are expected to improve detection accuracy of quarters/cows with inflammation(s) to be monitored or treated [13], particularly in local endangered populations. The recent European limitations on the antimicrobial use, in addition, have changed the routine of dairy farmers, making the analysis of SCC trend within lactation indispensable for a smart and correct application of selective dry-cow therapy protocol.

The DSCC phenotypic variability and relationship with SCC have never been investigated in Italian local breeds. Therefore, in the present study we investigated the effect of season, parity and stage of lactation on SCC and DSCC in Burlina and Alpine Grey. Subsequently, we evaluated if and how the udder health status identified with different combinations of SCC and DSCC can affect milk yield and composition.

## 2. Materials and Methods

### 2.1. Data Editing

Information on individual milk samples was retrieved from the official routine milk testing database of the Breeders Association of the Veneto Region (ARAV, Vicenza, Italy). Routine milk testing is carried out every 4–5 weeks and include registration of daily milk yield (MY, kg/d). The sampling period covered 24 months, from January 2019 to December 2020 and included all year-round calving herds. The infrared-predicted traits included, protein, casein, and lactose content (%), and urea (mg/dL) and beta-hydroxybutyrate concentration (BHB, mmol/L) determined using the Combifoss 7 DC (Foss, Hillerød, Denmark). SCC and DSCC were obtained by flow cytometry as described in [6].

The casein index was calculated as the ratio between casein and protein. To achieve a normal distribution of the data, SCC was transformed to SCS through the formula of Ali and Shook [14]: SCS = 3 + log2(SCC/100,000), and score of DSCC, i.e., DSCS, was calculated using the same formula starting from DSCC expressed in cells/mL, which was obtained as: DSCC (cells/mL) = SCC (cells/mL) × DSCC (%). Finally, following Benedet et al. [15], the BHB was log10-transformed. According to Schwarz et al. [7], the good separation index (GOSE) of Combifoss 7 DC provides information about reliability of the measured SCC and DSCC. For this reason, 168 test-day records lacking good separation (GOSE = 0) were discarded. Moreover, only samples with SCC ≥ 10,000 and ≤ 5,000,000 cells/mL analyzed within 5 d from sampling were considered. This ensured the presence of highly reliable SCC and DSCC [6]. Records not belonging to cows between 5 and 305 days in milk (DIM) or which were of a parity greater than 9 were discarded. Similarly, cows with less than 3 test-day records within each lactation and herd-test-dates with less than 3 cows were also discarded. Values of MY and fat, protein, and lactose content deviating more than 3 standard deviations from the mean were considered as missing.

**Figure 1:**
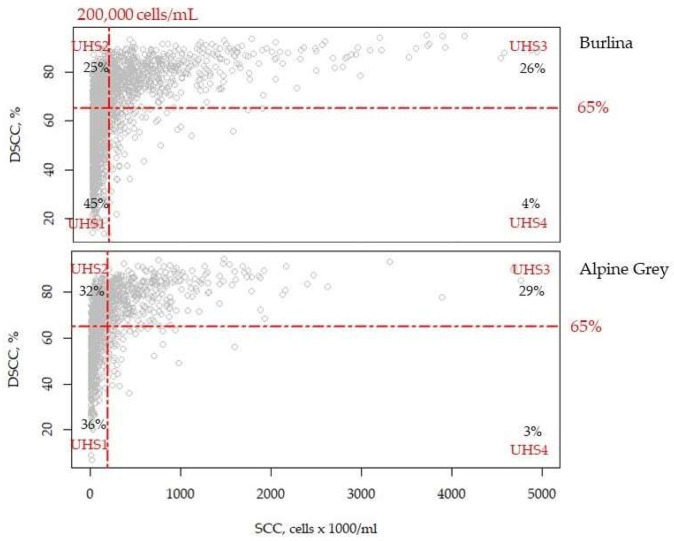
Distribution of milk test-day records across the four udder-health status (UHS) groups identified in the two breeds by mean of specific cut-offs (in red). The amount (%) of milk test-day records is reported in each quadrant (in black). Records were classified according to somatic cell count (SCC) and differential somatic cell count (DSCC, combined proportion of polymorphonuclear neutrophils and lymphocytes out of the total SCC) as follows: UHS1 if SCC ≤ 200,000 cell/mL and DSCC ≤ 65%, UHS2 if SCC ≤ 200,000 cell/mL and DSCC > 65%, UHS3 if SCC > 200,000 cell/mL and DSCC > 65%, and UHS4 if SCC > 200,000 cell/mL and DSCC ≤ 65%.

The final dataset included 3978 test-day records from 212 Alpine Grey and 426 Burlina cows located in 20 and 16 herds, respectively. Following Schwarz et al. [7], test-day records were categorized based on SCC and DSCC into four udder health status (UHS) groups (Figure 1). Briefly, we considered cows with SCC ≤ 200,000 cells/mL and DSCC ≤ 65% as healthy (UHS1), and cows with SCC ≤ 200,000 cells/mL and DSCC > 65% suspicious of mastitis (UHS2). SCC > 200,000 cells/mL and DSCC > 65% were the inclusion criteria for the UHS3 group. Based on the most accredited interpretation, finally, SCC > 200,000 cells/mL and DSCC ≤ 65% (UHS4) identified animals with chronic intramammary infection(s).

### 2.2. Statistical Analysis

Data manipulation, editing, and analysis were carried out in R software v. 4.1.2 [16]. In the first step, the analysis of variance was performed for SCS, DSCC (%) and DSCS as dependent variables through the following mixed linear model:

y*_ijklmnop_* = µ + B*_i_* + S*_j_* + P*_k_* + D*_l_* + T*_m_* + (B × S)*_ij_* + (B × P)*_ik_* + (B × D)*_il_* + C*_n_* + H*_o_*(B*_i_*) + e*_ijklmnop_* (1)

where y*_ijklmnop_* is the dependent variable; µ is the overall mean; B*_i_* is the fixed effect of the *i*th breed (*i* = Alpine Grey and Burlina); S*_j_* is the fixed effect of the *j*th season of sampling (*j* = 4 season: December to February, March to May, June to August, and September to November); P*_k_* is the fixed effect of the *k*th parity (*k* = 1, 2, 3, 4, and ≥5, with the last containing data up to parity 9); D*_l_* is the fixed effect of the lth stage of lactation (*l* = 6 classes of 50 d each); T*m* is the day of analysis calculated as the difference between the sampling date and the milk analysis date (*m* = 0 to 5 days); (B × S)*_ij_* is the fixed interaction effect between breed and season of sampling; (B × P)*_ik_* is the fixed interaction effect between breed and parity; (B × D)_*il*_ is the fixed interaction effect between breed and stage of lactation; C_*n*_ is the random effect of the _*n*_th cow (n = 691) ~ N(0, σ^2^_C_), where σ^2^_C_ is the cow variance; H_*o*_(B_*i*_) is the random effect of the *_o_*th herd-test-date (*o* = 344) nested within breed ~N(0, σ^2^_H(B)_), where σ^2^_H_ is the herd-test-date variance; and e*_ijklmnop_* is the random error ~N(0, σ^2^_e_), where σ^2^_e_ is the residual variance. Model diagnostics were checked through analysis of distribution, variance homogeneity, and independence of residuals.

Subsequently, four combinations of SCC and DSCC were used to include UHS as independent variable in a further mixed linear model and estimate its impact on MY and major milk composition traits:

y*_ijklmnopq_* = µ + B*_i_* + S*_j_* + P*_k_* + D*_l_* + U*_m_* + T*_n_* + (B × S)*_ij_* + (B × P)*_ik_* + (B × D)*_il_* + (B × U)*_im_* + C*_o_* + H*_p_*(B*_i_*) + e*_ijklmnop_* (2)

where all the effects, except for UHS group (U*_m_*), were the same included in Equation (1). The UHS group (Figure 1) was included as main effect and in interaction with breed. Multiple comparisons of least squares means were performed using the Bonferroni adjustment with significance at *p* ≤ 0.05, unless otherwise stated.

## 3. Results

### 3.1. SCS and DSCC Variability

The raw means of daily production and gross composition traits of the two breeds were similar, while the average SCS, DSCC, and DSCS were greater for the Alpine Grey than for the Burlina (Table 1). The coefficient of variation (CV), calculated as the ratio of the standard deviation to the mean, was 27% for urea in Alpine Grey and 30% in Burlina; BHB was more variable in the former (CV = 34%) than in the latter breed (CV = 28%). Negative values of BHB refer to samples with low concentrations before log-transformation.

As regards Pearson’s correlations (Table 2), SCS was strongly positively correlated with both DSCC (0.66) and DSCS (0.99) and inversely with MY, lactose, and casein-index, similarly to DSCS. DSCC presented not significant coefficients with protein and casein and, in general, urea concentration was not significantly correlated with the udder health traits studied with. In both breeds most of the test-day records presented SCC < 200,000 cells/mL (Figure 1). 36 and 45% of the data of Alpine Grey and Burlina belonged to UHS1, and 31 and 26% to UHS2 (Figure 1). The udder health group with the lowest frequency (<4%) was UHS4 (Figure 1).

**Figure 2:**
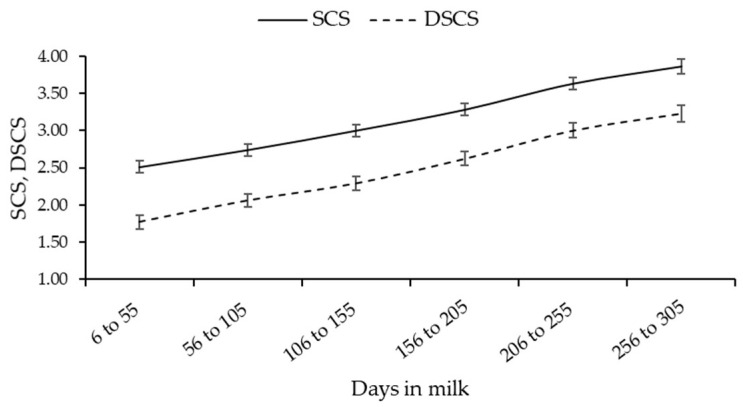
Least squares means of SCS (somatic cell score) and DSCS (log2-transformation of number of polymorphonuclear neutrophils and lymphocytes in milk (cells/mL)) across lactation.

Both least squares means of SCS and DSCS were similar in the two breeds (Table S1); however, the estimated DSCC was greater in Alpine Grey (66.2 ± 0.8%) than Burlina (63.2 ± 0.6%). Although lactation stage was significant for both SCS and DSCS (Figure 2), the interaction between lactation stage and breed was not (Table S1).

**Figure 3:**
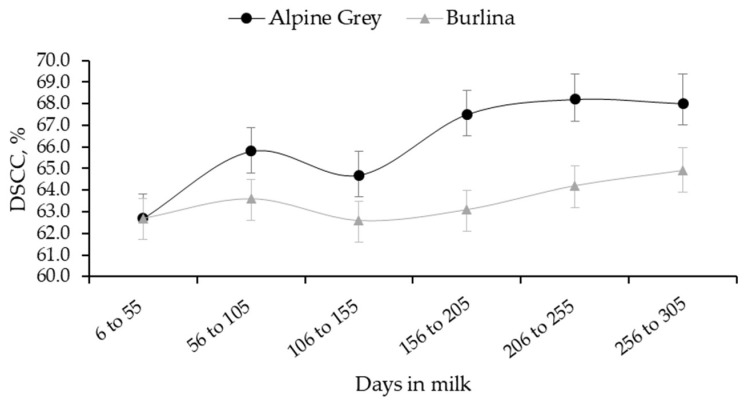
Least squares means of DSCC (combined proportion (%) of polymorphonuclear neutrophils and lymphocytes out of the total somatic cell count (cells/mL)) for the interaction between lactation stage and breed.

The lowest SCS and DSCS were observed in early lactation, whereas the greatest at the end. The two breeds showed different patterns of DSCC (*p* < 0.05) throughout DIM (Figure 3), with Alpine Grey having the minimum in early lactation (62.5 ± 1.1%), a peak corresponding with the MY lactation peak (Figure 3; 66.2 ± 1.1%) and a subsequent drop between 106 and 155 DIM followed by a second increase and stabilization. On the contrary, least squares means of DSCC were similar across lactation stages in Burlina cows, with a mild peak in correspondence of the MY lactation peak (Figure 3).

SCS, DSCC, and DSCS were significantly affected by parity and its interaction with breed (Table S1). SCS steadily increased with cow’s parity (Table 3) and the last parity class presented the greatest DSCS in both breeds (Table 3).

**Figure 4:**
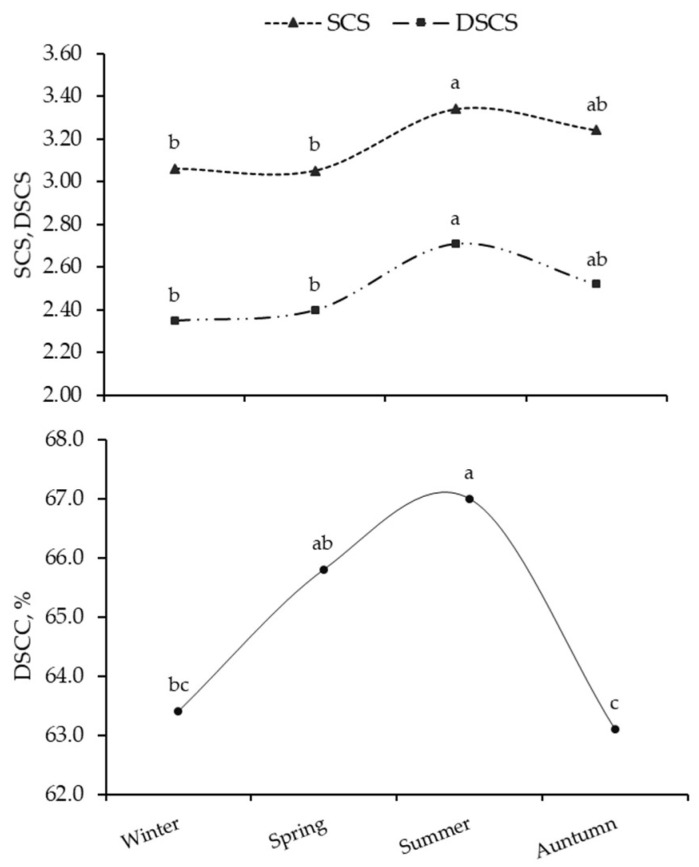
Least squares means of udder health traits across seasons of sampling. SCS, somatic cell score; DSCC, combined proportion (%) of polymorphonuclear neutrophils and lymphocytes out of the total somatic cell count (cells/mL); DSCS, log2-transformation of number of polymorphonuclear neutrophils and lymphocytes in milk (cells/mL). Values with different superscripts within udder health trait are significantly different (*p* ≤ 0.05).

Season of sampling—but not its interaction with the breed—affected the SCS, DSCC, and DSCS variability (Table S1, Figure 4). Least squares means were generally the greatest in summer (SCS = 3.34 ± 0.09; DSCS = 2.71 ± 0.10), except for DSCC whose seasonal trend looked different, being characterized by a single peak in summer (66.6 ± 0.6%) and a drop in autumn (63.3 ± 0.7%).

### 3.2. Effect of Udder Health

**Figure 5:**
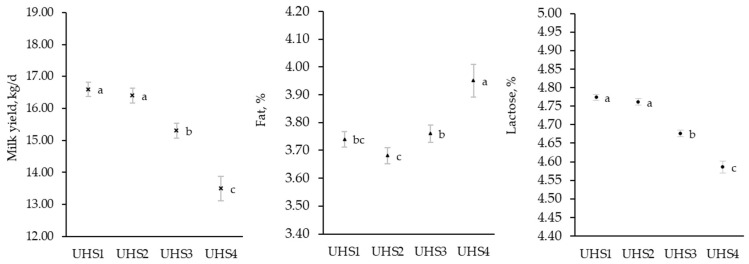
Least squares means of milk yield, fat, and lactose content in different udder health status groups: Milk test-day records were classified according to somatic cell count (SCC) and differential somatic cell count (DSCC) as follows: UHS1 if SCC ≤ 200,000 cell/mL and DSCC ≤ 65%, UHS2 if SCC ≤ 200,000 cell/mL and DSCC > 65%, UHS3 if SCC > 200,000 cell/mL and DSCC > 65%, and UHS4 if SCC > 200,000 cell/mL and DSCC ≤ 65%. Values with different superscripts within trait are significantly different (*p* ≤ 0.05).

In both breeds, the UHS significantly affected daily MY and composition. The interaction between UHS and breed was in fact significant for protein and casein, BHB, and urea, whereas there was only a tendency for the casein-index (Table S2). Least squares means of Burlina MY (16.10 ± 0.24 kg/d) was greater compared to Alpine Grey (15.30 ± 0.32 kg/d) and MY differed according to the UHS group (Figure 5).

The greatest daily MY was in fact observed in UHS1 (17.10 ± 0.21 kg/d), i.e., when SCC ≤ 200,000 cells/mL and DSCC ≤ 65%. This estimate, however, was similar to that of UHS2. Overall, cows presenting SCC > 200,000 cells/mL had a sharp drop in MY, in fact, the lowest production (13.40 ± 0.41 kg/d) was observed in the UHS4 group.

UHS significantly affected part of the composition traits (Figure 5). The greatest lactose content was observed in groups with SCC ≤ 200,000 cell/mL, i.e., UHS1 (4.78 ± 0.01%) and UHS2 (4.77 ± 0.01%), while the lowest content belonged to UHS4 records (4.58 ± 0.02%). The lowest and the greatest fat content was observed in UHS2 (3.67 ± 0.03%) and UHS4 (3.98 ± 0.06%), respectively. The interaction between UHS and breed was significant for protein and casein content and casein index (Table 4).

In both Alpine Grey and Burlina, UHS1 and UHS2 presented the lowest protein content, whereas the greatest was observed in UHS4 (Alpine Grey, 3.53 ± 0.04%; Burlina 3.60 ± 0.03%). When comparing the two extreme classes, UHS4 and UHS1, the relative reduction in protein was greater in Burlina than in the other (−0.21 and −0.12%, respectively). The same was observed for casein content of Burlina milk (Table 4). In Alpine Grey breed the numerically greatest estimate of casein was found in UHS3, which was not significantly different from UHS1 and UHS2. The lowest casein-index was observed for UHS4. The greatest casein-index estimate was calculated for groups with SCC < 200,000 cells/mL, i.e., UHS1 and UHS2.

As regards the milk BHB (Table 4), indicator of negative energy balance, all breeds had the greatest estimates in UHS4, with the score estimated at −1.22 ± 0.06 and −1.16 ± 0.04 for Alpine Grey and Burlina, respectively. In Burlina cows, milk BHB of UHS3 was similar to that of UHS4. In general, the lowest BHB was observed for records with SCC ≤ 200,000 cell/mL, i.e., UHS1 and UHS2, and the relative increase in BHB was greater in Alpine Grey than Burlina. Finally, the effect of UHS on the urea concentration was different in the two breeds: in Alpine Grey it ranged from 23.40 to 24.00 mg/dL with no differences among UHS groups, whereas in Burlina the lowest estimate was found in UHS3 whereas the highest in UHS4 and UHS1.

## 4. Discussion

Compared to the endangered Burlina breed, the Alpine Grey is present in a greater geographical area of the Northern Italian Alps. At the present, Italian Alpine Grey population consists in 17,548 officially registered heads in 1784 farms [2]. The main breeding goal in this breed is to improve the aptitude for milk and meat production in parallel, maintaining the typical morphological characteristics [1]. On the other hand, Burlina is only present in 39 farms, and only in the mountain areas of the Veneto region involved in a specific conservation plan. In 2020 this breed accounted for 1060 heads [2]. From a genetic standpoint, the interest is to preserve Burlina in purity, focusing on the inbreeding level for motivations related to biodiversity [3]. Local breeds are often characterized by robustness, optimal fertility, and longevity. Indeed, Burlina and Alpine Grey have a calving-conception interval average of 91 and 99 days, respectively, which are significantly lower than that reported for Holstein Friesian (127 days) [17]. Moreover, the frequency of abortions is very low (≤0.2%) and the frequency of twin calving is greater in Alpine Grey (3.11%) than Burlina (0.26%) [17]. The age at first calving of these local breeds is higher than Holstein Friesian; however, the average number of lactations is greater (3.34 for Alpine Grey and 3.31 for Burlina) [17]. Despite the type of breeding of Alpine Grey and Burlina, calvings are distributed through the year, with slightly higher percentages in autumn [17].

### 4.1. SCS and DSCC Variability

Beyond being influenced by breed, udder health is first and foremost affected by management. Local breeds are generally confined to mountain areas where the traditional rearing system is the tie-stall barn with summer grazing. Different studies [18,19] have demonstrated that SCS of Alpine Grey, Holstein Friesian, and Simmental are similar and greater than SCS of Brown Swiss. Gottardo et al. [18] and Visentin et al. [19] reported estimates of 2.59 and 2.61 for SCS in Alpine Grey cows farmed in South Tyrol. Regarding Burlina breed, Penasa et al. [20] reported an average SCS of 3.56. In the same breed, Niero et al. [21] observed an average of 3.13. SCS tends to be greater in Burlina than Holstein Friesian cows [22]. In the present study average SCS was numerically greater in Alpine Grey than Burlina. However, DSCC was significantly greater in Alpine Grey. To our knowledge, no studies have explored DSCC variability in local breeds such as Burlina and Alpine Grey so far. In terms of raw means, DSCC of Burlina and Alpine Grey was greater if compared with the results of Bobbo et al. [23] that found a raw means of 61.8% in Holstein Friesian, 60.8% in Brown Swiss and 61.1% in Simmental cows. If compared to the local Rendena breed (DSCC = 66.6%), Burlina DSCC is lower [23].

In lactating cows, the number of mammary gland secretory cells increases with parity/age, resulting in a progressive lifetime increase in MY. At the same time, however, this exposes cows to major stress, compromised immune response and elevated sensitivity to intramammary infection [7,24]. This is in line with results in Table 3, which suggested that udder health is generally worse in older cows. Our results showed that SCS and DSCS had similar trends across DIM, with the lowest SCS in early lactation stages, likely due to a dilution effect. After the MY peak, SCS steadily increased until the end of lactation. On the contrary, DSCC, like SCC, peaked together with MY between 56 and 105 DIM, but was high even in late lactation. This demonstrates that the fractions of SCC changes along DIM, with the amount of polymorphonuclear neutrophils increasing towards the end of lactation [25,26]. According by Schwarz et al. [7], SCC and SCS increased in the warm season due to exposure to heat, reduced feed intake and greater susceptibility to diseases; in addition, higher temperatures and greater humidity in the summer are optimal conditions for proliferation of environmental (bedding) bacteria [27].

### 4.2. Udder Health Status Group and Daily Production and Composition

MY of Alpine Grey and Burlina were similar and, in accordance with Schwarz et al. [8], were influenced by UHS. When SCC increases, the daily MY decreases due to intramammary infections. Mastitis pathogens and agents released during immune response are responsible for damages of udder tissue, which is particularly evident when mastitis is subclinical or recurrent [28,29]. This might explain the lower MY observed for UHS4 (‘chronic’; [7]) compared to the others. Cows with high SCC show lower MY followed by a concentration of certain solids, namely fat and protein. Despite this, mastitis is known to cause unfavorable changes in specific detailed fractions, e.g., casein content reduction and lipolysis occurrence. Intramammary infection causes an influx of leukocytes from the blood, with alteration of the osmotic equilibrium in the alveoli and thus modification of milk composition [5].

In the study of Bobbo et al. [23], the concentration effect was responsible for the great fat content found in cows with chronic mastitis (here UHS4). In the same study, cows suspicious of intramammary infection (here UHS2) were characterized by the lowest milk fat content probably due to the lipolytic activity stimulated by neutrophils recruitment [30]. The proteolytic activity impairs the casein fraction, and with SCC/mastitis the proteolytic activity becomes elevated, thereby reducing the favorable proteins that are synthesized within the mammary gland (α-casein, β-casein, α-lactalbumin, and β-lactoglobulin). This creates grounds for increased transfer of protein fractions from blood such as serum albumin and immunoglobulins [31]. The greatest protein and casein contents were found in group UHS4, where MY was the lowest leading to a concentration of some solids. For the reason explained above, the casein index was expected to be the lowest in UHS4. This agrees with Mariani et al. [32] who observed the lowest casein index in cows with simultaneously high SCC and low DSCC.

As regards lactose, results are in agreement with Costa et al. [29]. Overall, cows with high SCC presented lower lactose content due to leakage caused by the compromised alveolar epithelial integrity. Mastitis creates damages at the epithelial level in the alveolar structures; all the substances released during and after mammary gland tissues inflammation, such as bacteriostatic and bactericidal factors, disrupt tight junctions of the basal membrane that separates blood and milk [29,33,34]. Even if deeper investigations are needed, lactose losses are generally expected to be greater in cows/quarters suffering from a condition of chronic mastitis.

The association between cow’s udder health and infrared-predicted milk BHB has only been marginally investigated in the literature. The present study showed that milk BHB was maximum in the UHS4 group. Moyes et al. [35] observed that udder inflammation was associated with an increase in milk BHB, indicating that animals with consistently high SCC throughout their lifetime could be more susceptible to metabolic disorders and severe negative energy balance.

Milk urea concentration was the lowest in UHS3 group. This is, at least partly, in line with Mariani et al. [32] who observed a lower concentration of urea in milk of Holstein-Friesian and Simmental cows that presented high values for both DSCC and SCC. Pegolo et al. [36] did not report significant associations between milk urea and SCC at different levels of DSCC in Holstein Friesians. Milk urea is usually adopted to monitor protein utilization and nitrogen efficiency at herd level for management purpose (bulk milk). A large concentration of urea in individual milk is instead indicative of inefficient nitrogen utilization/protein metabolism due to either unbalanced protein content of feed (e.g., temporary excess) or cow-related physiological factors. In fact, urea in milk is not influenced by breed but rather by the interaction of multiple environmental factors.

### 4.3. DSCC and SCC in Practise

The UHS of cows influences the economic profit of the farmer both directly and indirectly. Findings of this study demonstrate that cows with high SCC (UHS3 and UHS4) tend to produce less [37] and to present a deteriorated milk quality. Within the supply chain, high SCC in bulk milk leads to penalties for the farmers, particularly in Italy where some milk components important for cheesemaking are considered in the milk payment system [38]. Even when the milk is processed directly at the farm, the consequences of UHS3 and UHS4 negatively affect the milk and cheese yield (e.g., lower casein index). In addition to this, costs for any veterinary treatment during the lactation and/or the dry period must be accounted for while also an increased culling rate could arise [5]. Ideally, we will need further validation data to confirm whether DSCC I) significantly improves diagnostic accuracy of mastitis or II) is useful for the identification of cows needing treatment at dry-off. This would lead to a decrease in antibiotic use and costs at farm-level.

## 5. Conclusions

In the present study we demonstrated how the different milk somatic cell fractions were differently affected bybreed (Apline Grey and Burlina), stage of lactation, parity, and season of sampling. Primiparous showed lower SCC and DSCC compared to pluriparous and udder health seemed in general worse in the summer period. Moreover, findings releval that udder health status can affect MY and milk composition in local cattle breeds. This is the first study investigating proxies for udder health in Italian local breed. Our results highlight that udder health of less cosmopolitan/autochthonous cows can be monitored through official milk analyses exploiting various proxies measurable in milk. To improve udder health, the introduction of mastitis recording and monitoring plans is advisable in local as in cosmopolitan breeds and may allow the set-up of dedicated genetic strategies. The low productivity of dual purpose/local breeds compared to the highly selected populations must be compensated through maximization of their resistance to disease, longevity, functionality, and efficiency, with a positive indirect reduction in biodiversity loss.

## Figures and Tables

**Table 1:** Overview of descriptive statistics of milk test-day records for each breed.

Trait ^1^	Burlina				Alpine Grey			
	Mean	SD	Min.	Max.	Mean	SD	Min.	Max.
Milk yield, kg/d	16.99	6.70	1.80	37.3	16.99	5.85	2.20	33.6
Fat, %	3.80	0.73	1.45	6.22	3.57	0.63	1.57	5.59
Protein, %	3.35	0.41	2.21	4.63	3.37	0.39	2.43	4.55
Casein, %	2.62	0.33	1.64	3.66	2.65	0.32	1.81	3.63
Casein- index	0.78	0.02	0.69	0.84	0.78	0.02	0.69	0.83
Lactose, %	4.72	0.23	3.92	5.31	4.80	0.21	4.06	5.49
SCS	3.06	1.87	−0.32	8.63	3.13	1.82	−0.32	8.57
DSCC, %	63.5	16.4	13.7	95.0	66.8	14.3	7.2	94.1
DSCS	2.34	2.16	−3.15	8.45	2.51	2.10	−3.05	8.40
BHB	−1.30	0.36	−2.00	−0.24	−1.51	0.51	−3.00	−0.54
Urea, mg/dL	23.55	6.97	6.6	44.8	23.59	6.46	5.90	43.6

^1^ SCS, somatic cell score; DSCC, combined proportion (%) of polymorphonuclear neutrophils and lymphocytes out of the total somatic cell count (cells/mL); DSCS, log2-transformation of number of polymorphonuclear neutrophils and lymphocytes in milk (cells/mL); BHB, log10-transformed beta-hydroxybutyrate concentration (mmol/L).

**Table 2:** Pearson’s correlations ^1^ (*p* ≤ 0.05) between milk yield and composition traits ^2^.

Trait ^2^	SCS	DSCC	DSCS
DSCC, %	0.66		
DSCS	0.99	0.77	
Milk yield, kg/d	−0.23	−0.08	−0.22
Fat, %	0.08	−0.08	0.05
Protein, %	0.20	−0.02 ^ns^	0.17
Casein, %	0.16	−0.03 ^ns^	0.13
Casein index	−0.19	−0.04	−0.17
Lactose, %	−0.43	−0.13	−0.39
BHB	0.08	0.03	0.08
Urea, mg/dL	−0.01 ^ns^	−0.01 ^ns^	−0.01 ^ns^

^1^ ns, not significant. ^2^ SCS, somatic cell score; DSCC, combined proportion (%) of polymorphonuclear neutrophils and lymphocytes out of the total somatic cell count (cells/mL); DSCS, log2-transformation of number of polymorphonuclear neutrophils and lymphocytes in milk (cells/mL); BHB, log10-transformed beta-hydroxybutyrate concentration (mmol/L).

**Table 3:** Least squares means ^1^ of udder health traits ^2^ for the interaction between parity and breed. Values with different superscripts within row are significantly different (*p* ≤ 0.05).

Trait	Breed	Parity				
		1	2	3	4	≥5
SCS	Alpine Grey	2.53 ^c^	2.48 ^c^	3.10 ^b^	2.87 ^bc^	3.93 ^a^
	Burlina	2.50 ^c^	2.85 ^b^	2.94 ^b^	3.25 ^ab^	3.44 ^a^
DSCC	Alpine Grey	64.8 ^b^	63.4 ^b^	66.7 ^ab^	63.4 ^b^	71.0 ^a^
	Burlina	62.5 ^ab^	62.3 ^ab^	61.0 ^b^	64.2 ^ab^	65.8 ^a^
DSCS	Alpine Grey	2.03 ^bc^	1.95 ^c^	2.65 ^b^	2.38 ^bc^	3.58 ^a^
	Burlina	1.95 ^c^	2.28 ^bc^	2.33 ^bc^	2.73 ^ab^	2.96 ^a^

^1^ Standard errors ranged from 0.10 to 0.17 for SCS, 0.93 to 1.42 for DSCC, and 0.12 to 0.20 for DSCS. ^2^ SCS, somatic cell score; DSCC, combined proportion (%) of polymorphonuclear neutrophils and lymphocytes out of the total somatic cell count (cells/mL); DSCS, log2-transformation of number of polymorphonuclear neutrophils and lymphocytes in milk (cells/mL).

**Table 4:** Least squares means ^1^ of protein, casein, casein-index, BHB ^2^ and milk urea concentration for the interaction between udder health status group ^3^ and breed. Values with different superscripts within row are significantly different (*p* ≤ 0.05).

Trait	Breed	UHS1	UHS2	UHS3	UHS4
Protein, %	Alpine Grey	3.41 ^b^	3.41 ^b^	3.48 ^a^	3.53 ^a^
	Burlina	3.39 ^c^	3.35 ^d^	3.44 ^b^	3.60 ^a^
Casein, %	Alpine Grey	2.69 ^b^	2.70 ^ab^	2.73 ^a^	2.74 ^a^
	Burlina	2.66 ^b^	2.63 ^c^	2.69 ^b^	2.80 ^a^
Casein-index	Alpine Grey	0.787 ^a^	0.789 ^a^	0.782 ^b^	0.777 ^c^
	Burlina	0.786 ^a^	0.786 ^a^	0.781 ^b^	0.777 ^b^
Urea, mg/dL	Alpine Grey	24.00 ^a^	23.70 ^a^	23.40 ^a^	23.50 ^a^
	Burlina	23.30 ^a^	23.20 ^ab^	22.60 ^b^	23.50 ^ab^
BHB^2^	Alpine Grey	−1.58 ^c^	−1.54 ^bc^	−1.47 ^b^	−1.22 ^a^
	Burlina	−1.31 ^b^	−1.32 ^b^	−1.22 ^a^	−1.16 ^a^

^1^ Standard errors ranged from 0.02 to 0.04 for protein and casein, 0.0001 to 0.002 for casein-index, 0.45 to 0.73 for urea and 0.03 to 0.06 for BHB. ^2^ BHB, log10-transformed beta-hydroxybutyrate concentration (mmol/L). ^3^ Milk test-day records were classified according to somatic cell count (SCC) and differential somatic cell count (DSCC) as follows: UHS1 if SCC ≤ 200,000 cell/mL and DSCC ≤ 65%, UHS2 if SCC ≤ 200,000 cell/mL and DSCC > 65%, UHS3 if SCC > 200,000 cell/mL and DSCC > 65%, and UHS4 if SCC > 200,000 cell/mL and DSCC ≤ 65%.

## Data Availability

The datasets generated during and/or analyzed during the current study are available from the corresponding author on reasonable request.

## Supplementary Materials

The following supporting information can be downloaded at: https://www.mdpi.com/article/10.3390/ani13071249/s1, Table S1: F-values and significance of fixed effects in Equation (1) for the traits recorded in Alpine Grey and Burlina cows; Table S2: F-values and significance of fixed effects in Equation (2) for the traits recorded in Alpine Grey and Burlina cows.
