# Supplementary material for: Total and Differential Somatic Cell Count in Italian Local Cattle Breeds: Phenotypic Variability and Effect on Milk Yield and Composition
Source: Animals (Basel). 2023 Apr 4;13(7):1249. doi: 10.3390/ani13071249 (PMC10093597; doi:10.3390/ani13071249)
Supplement: Supplementary file 1 [file animals-13-01249-s001.zip › animals-2282963-supplementary.pdf]

SUPPLEMENTARY MATERIALS

Total and Differential Somatic Cell Count in Italian Local Cattle Breeds: Phenotypic Variability and Effect on Milk Yield and Composition

Silvia Magro [1,*], Angela Costa [2] and Massimo De Marchi [1]

[1] Department of Agronomy, Food, Natural Resources, Animals and Environment, University of Padova, 35020 Padova, Italy; massimo.demarchi@unipd.it
[2] Department of Veterinary Medical Sciences, Alma Mater Studiorum University of Bologna, 40064 Bologna, Italy; angela.costa2@unibo.it
* Correspondence: silvia.magro.1@phd.unipd.it

Table S1. F-values and significance[1] of fixed effects in [Eq. 1] for the udder health traits[2] recorded in milk of Alpine Grey and Burlina cows.

Trait	Breed	Lactation stage	Parity	Season	Day of analysis	Lactation stage x breed	Parity x breed	Season x breed
SCS	ns	66.84 ***	22.76 ***	9.74 ***	3.69 **	ns	5.55 ***	ns
DSCC	9.16 **	7.37 ***	6.75 ***	14.90 ***	4.86 ***	2.29 *	2.83 *	ns
DSCS	ns	59.58 ***	20.83 ***	8.53 ***	3.93 **	ns	5.54 ***	ns

[1]****P*<0.001; ***P*<0.01; **P*<0.05; ns = not significant.

[1]SCS, somatic cell score; DSCC, combined proportion (%) of polymorphonuclear neutrophils and lymphocytes out of the total somatic cell count (cells/ml); DSCS, log_2-transformed combined number of polymorphonuclear neutrophils and lymphocytes in milk (cells/mL).

Table S2. F-values and significance[1] of fixed effects in [Eq. 2] for the milk traits[2] recorded in Alpine Grey and Burlina cows.

Trait	Breed	Lactation stage	Parity	Season	Udder health group	Day of analysis	Lactation stage x breed	Parity x breed	Season x breed	Udder health group x breed
Milk yield, kg/d	4.22 *	398.28 ***	39.27 ***	4.22 ***	45.98 ***	ns	3.11 **	ns	38.69 ***	ns
Fat, %	14.80 ***	54.27 ***	7.63***	6.17 ***	9.55 ***	9.59 ***	2.33 *	ns	4.02 **	ns
Protein, %	ns	375.20 ***	2.45 *	68.65 ***	23.00 ***	2.61 *	3.35 **	ns	7.59 ***	2.19 †
Casein, %	ns	420.04 ***	ns	89.01 ***	11.06 ***	3.28 **	3.67 **	ns	5.54 ***	3.45 *
Casein index	ns	46.71 ***	4.07 ***	41.68 **	47.20 ***	12.11 **	ns	ns	3.92 ***	2.18 †
Lactose, %	60.43 ***	40.86 ***	46.66 ***	62.83 ***	98.98 ***	7.61 ***	2.44 *	2.66 *	15.67 ***	ns
BHB	59.84 ***	12.55 ***	4.27 **	15.77 ***	19.21 ***	7.30 ***	4.15 ***	ns	7.60 ***	2.31 †
Urea, mg/dL	ns	420.03 ***	ns	89.01 ***	11.06 ***	3.27 **	3.67 **	ns	5.54 **	3.45 *

[1]****P*<0.001; ***P*<0.01; **P*<0.05; †*P*<0.10; ns = not significant.

[2]BHB, log_{10}-transformed beta-hydroxybutyrate concentration (mmol/L).
